# Impact on the Quality of Erections after Completing a Low-Intensity Extracorporeal Shock Wave Treatment Cycle on a Group of 710 Patients

**DOI:** 10.1155/2017/1843687

**Published:** 2017-12-21

**Authors:** Héctor A. Corredor Ayala, José Pablo Saffon Cuartas, Diana Cerquera Cleves

**Affiliations:** ^1^Grupo de Investigación Clínica, Boston Medical Group, Cra. 10 No. 97a-13 of 308, Bogotá, Colombia; ^2^Clinical Sexology, Grupo de Investigación Clínica, Boston Medical Group, Cra. 10 No. 97a-13 of 308, Bogotá, Colombia; ^3^Universidad Nacional de Colombia, Cra. 45 No. 26-85, Bogotá, Colombia

## Abstract

**Objective:**

The aim of this study is to evaluate the response to low-intensity extracorporeal shock wave therapy in a group of patients with organic vascular erectile dysfunction.

**Materials and Methods:**

This is an observational retrospective study. The researchers reviewed 710 patients with a clinical diagnosis of organic vascular erectile dysfunction (ED) of more than 3-month duration from male sexual health clinics of the Boston Medical Group from 12 cities in Spain and 4 in Mexico. Patients received 5 outpatient shock wave therapy sessions. They were evaluated with the erection hardness score (EHS) before the first session (*n* = 710), at the end of the last session (*n* = 710), and one month after the last session (*n* = 412).

**Results:**

In the first examination, the EHS improved in 43.1% (306/710) of subjects compared to the baseline measurement and ability to penetrate increased from 26.8% to 44% (*p* < 0.0001). In the second examination, the ability to penetrate was 37.9%, lower than in the first (*p*=0.042) but higher than the baseline (*p*=0.0001).

**Conclusions:**

The results suggest that the shock wave therapy with or without concomitant treatments improved the quality of erections in patients with erectile dysfunction treated in specialised male sexual health clinics. This trial is registered with NCT03237143.

## 1. Introduction

Erectile dysfunction (ED) is defined as the persistent inability to attain or maintain an erection that is sufficient for satisfactory sexual intercourse until ejaculation or until the cessation of erotic stimulation. The pathophysiology of ED may be vasculogenic, neurogenic, anatomical, hormonal, drug-induced, and/or psychogenic [1, 2]. The Massachusetts Male Aging Study reported an overall prevalence of 52% in men aged 40 to 70, while the Cologne study estimated a prevalence of 19.2% in men between the ages of 30 and 80 with an age-related increase from 2.3% to 53.4%. It therefore has a significant negative impact on the quality of life of sufferers and their partners [3–5].

For the treatment of ED, recommendations for initial management include lifestyle changes that lead to heart-healthy habits [6, 7], to convince patients that all that is good for the heart is good for lovemaking; teaching healthy eating habits and cardiovascular exercise; minimising modifiable risk factors; and the detection and timely treatment of underlying chronic or acute pathologies associated with ED. The occurrence of erectile dysfunction is considered a cardiovascular warning sign (possibly associated with generalised endothelial dysfunction), which significantly increases the risk of cardiovascular disease, coronary disease, stroke, and generally an increased mortality risk. It therefore calls for a multidisciplinary approach to every patient who suffers from ED [7–9].

For symptomatic management of ED, the use of pharmacological therapies has been recommended, such as phosphodiesterase 5 inhibitors (PDE5Is); intracavernous, topical, or intraurethral vasodilators; and nonpharmacological treatments such as the use of vacuum pumps. However, although these therapies have been proven to be safe and effective, they do not alter the pathophysiology of erectile dysfunction or cardiovascular risk [10, 11].

Furthermore, it has been reported that low-intensity extracorporeal shock wave therapy (LI-ESWT), applied to the corpora cavernosa of patients with vascular ED, improves the quality of the erection by inducing angiogenesis mediated by intra- and extracellular mechanisms such as increased levels of nitric oxide synthase, nitric oxide, and vascular endothelial growth factors [12–15].

LI-ESWT has already been included in guidelines for the management of ED as a first-line therapeutic option [10]. The objective of this study is to evaluate the response to low-intensity extracorporeal shock wave therapy in a group of patients with organic vascular erectile dysfunction with ED symptoms for more than three months, who were treated at the BMG clinics.

## 2. Materials and Methods

Description of the cohort of patients treated between April 2014 and February 2015 at the Boston Medical Group male sexual health clinics of twelve cities in Spain (Madrid, Barcelona, Valencia, Seville, Coruña, Bilbao, Malaga, Zaragoza, Murcia, Alicante, Cordoba, and Jerez) and four in Mexico (Altavista, Reforma, Monterrey, and Naucalpan). Clinical records of patients over 18 years of age with a clinical diagnosis of organic vascular ED of more than three months and with an erection hardness score (EHS) [16, 17] of three or less were reviewed (an EHS of two or less indicates that the patient is not able to penetrate during intercourse, and an EHS of three or more indicates ability to penetrate). We excluded patients with ED caused by side effects from drugs or spinal cord injury; situational ED (obvious psychological origin); and lesions, loss of skin, or penile implant; and also excluded patients who did not adequately complete the LI-ESWT.

After the initial assessment and diagnosis consultation, each patient received a treatment of 5 sessions (1 session per week) of 20 minutes of LI-ESWT, carried out with the DUOLITH SD1 (Storz Tägerwilen, Switzerland). They received 3,000 impulses per session at 0.1 mJ/mm^2^, divided over six sites (four on the penis and two on the crura). All sessions were outpatient visits, and no anaesthetic was used. The EHS for all patients was measured at three stages: before the first session, at the end of the last session (5 weeks after the first session), and one month after the last session. When patients were receiving concomitant drug treatment for their dysfunction, they were asked to indicate their perception free of the effect thereof. During the examinations, known adverse effects of the treatment were investigated, such as pain, paresthesia, and skin lesions on the penis. All the clinical information was recorded by duly qualified physicians on a standardised form using centralised software on a secure server.

The age and the EHS values were described using median and interquartile range. The frequency of comorbidities and outcomes were expressed in absolute numbers and percentages, respectively. Differences in EHS values were compared using the Wilcoxon test for paired data as the variable was discrete, with nonnormal distribution. Additionally, using the ability to penetrate (EHS ≥ 3) as a dichotomous outcome, a raw and adjusted comparison of cointerventions was performed using the McNemar test and conditional logistic regression, respectively. All the hypothesis tests were two-tailed with a confidence level of 95%.

## 3. Results

A total of 710 patients were enrolled in the study, from twelve clinics in Spain and four clinics in Mexico. Baseline clinical characteristics of the patients in the study are shown in Table 1. Intracavernous or oral (sildenafil) drug therapy was administered in addition to LI-ESWT in 439 (61.8%) of the patients. No adverse reactions were recorded after treatment.

**Figure 1:**
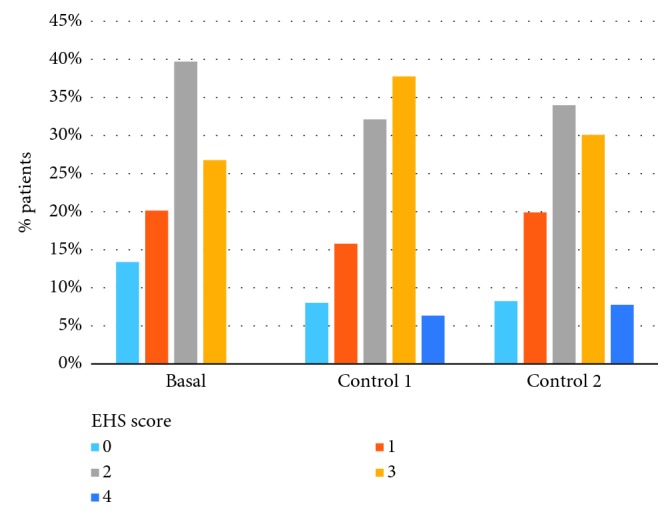
EHS progression during follow-up.

EHS progression at the different measurement times is shown in Figure 1. An initial decrease in the percentage of patients with low values (zero to two) and an increase for those with high values were observed in the first examination, with a partial reversal in the second examination. Although the median EHS did not vary in the three measurements (the score was two in all cases), the observed change in distribution was statistically significant when comparing the baseline measurement with each of the examinations (*p* < 0.0001) but not when comparing the examinations with each other (*p*=0.59).

In the first control after the treatment, 306 patients (43.1%) showed an improvement in their erections, 288 (40.6%) reported no change, and 116 (16.3%) worsened. The ability to achieve penetration increased from 26.8% to 44% (*p* < 0.0001).

In the second control, one month after the end of the treatment, the ability to achieve penetration was 37.9%, which is statistically higher than the baseline (*p*=0.0001) and lower than the previous measurement (*p*=0.042). 27.2% and 23.4% of patients showed an improvement in their erections compared to the previous examination and the baseline measurement, respectively. There were 298 (42%) patients at this follow-up stage due to failure to attend the appointment, of whom 137 (46%) had EHS values of 3 or 4 at the end of treatment.

Stratified analysis of the impact of LI-ESWT on the ability to achieve enough penis hardness to achieve penetration (EHS 3 or 4) based on other adjunctive therapies is shown in Table 2. The confidence intervals suggest that the observed difference in the percentages of improvement when comparing patients with or without additional treatment is not statistically significant. The raw ORs for the treatment to gain ability to penetrate after the first and second examinations were 3.36 (95% CI 2.46 to 4.68) and 2.2 (95% CI 1.49 to 3.31), respectively. The ORs adjusted for cointerventions were 5.7 (3.16 to 10.26) and 2.5 (1.28 to 4.88), respectively, indicating that shock wave therapy increases the likelihood that the patient regain their ability to penetrate regardless of other therapies received.

## 4. Discussion

This study involved the largest number of ED patients to have been treated with LI-ESWT published to date. The results show a significant improvement in erectile dysfunction, based on the erection hardness scale of achieving sufficient penetration in patients undergoing low-intensity shock wave therapy.

Patients received five sessions of LI-ESWT without requiring anaesthesia and with no evidence of complications; more than half of the sample received an additional drug therapy. During and after the procedure, three evaluations were carried out that showed favourable results, with an EHS improvement in patients undergoing treatment with respect to the baseline measurement, thus increasing the ability to penetrate. These results are equivalent to those reported in the literature in studies that applied similar methods [17, 18]. The width of the confidence intervals when assessing the impact of alternative drug therapies used by these patients suggests that the differences could not be clinically relevant.

The success of this therapy is based on its safety and ease of application, as well as it being the only therapy that seeks to modify the pathophysiology of the disease. Its effect on erectile dysfunction is due to the induction of different physiological components such as the nitric oxide synthase enzyme, endothelial growth factor, proliferating cell nuclear antigen, among other vasodilators, as well as the stimulation of stem cell migration which together lead to increased angiogenesis, thus improving the flow and the quality of erections [19, 20].

The strengths of the study include its large sample size, greater than any other found in the literature on LI-ESWT, and the retrospective data collection using standardised records, as well as it being a multicentre study. However, the shortcomings of the study include the considerable loss of patients to follow-up, which may affect the results, a short follow-up period, and the fact that a single outcome was evaluated, which is subjective. For this same reason, it was not possible to measure a change in the dosage of the concomitant medications. Additionally, due to the design of the study, part of the observed changes may be due to the Hawthorne effect, the placebo effect, regression to the mean, or other interventions.

For future studies, we propose that the best design to evaluate the effectiveness of the therapy is the placebo-controlled clinical trial; in addition, the evaluated parameters should be broadened in questionnaires on sexual satisfaction, quality of life, and more specific and objective outcomes such as blood flow in the penile Doppler ultrasound. Moreover, each LI-ESWT technology provider has a different application protocol, and no studies comparing these treatment regimens yet exist.

A striking finding was the general decline in the benefits after a month of treatment, raising the possibility of prolonging therapy for better results. In LI-ESWT studies in animals, it has been shown that local elevation of the aforementioned vascularisation and cell regeneration factors remain in the tissue for a period of approximately 8 weeks, and their greatest expression occurs around the first month [19], which opens the door for new studies to test this hypothesis.

## 5. Conclusion

LI-ESWT applied to the corpora cavernosa showed a statistically significant improvement in the quality of erections in ED patients with or without oral or intracavernous treatment, without side effects. The improvement effect diminished slightly in the final assessment.

## Figures and Tables

**Table 1:** Baseline characteristics of the study group.

Variable	Value,^∗^ median (range)/frequency (percent)
Age (years)	58 (24–83)
Baseline EHS	
** **0	95 (13.4)
** **1	143 (20.1)
** **2	282 (39.7)
** **3	190 (26.8)
Smokers	266 (37.5)
Patients in an established relationship	495 (69.7)
Previous treatment	430 (60.6)
Duration of dysfunction (years)	3.9 (0–50)
*Medical history (relevant diagnosis to ED)*	
Diabetes I	16 (2.3)
Diabetes II	140 (19.7)
Dyslipidemia	233 (32.8)
Hypertension	228 (32.1)
Hypertriglyceridemia	44 (6.2)
Acute myocardial infarction	19 (2.7)
Malignant prostate disease (in remission)	3 (0.4)
Prostatitis	7 (1)
Benign tumour of the prostate	35 (4.9)
Kidney transplant	2 (0.3)
Sexually transmitted diseases	4 (0.5)
Cerebrovascular accident	6 (0.8)
Peyronie's disease	7 (1)
HIV infection	4 (0.6)
Heart surgery	24 (3.4)
Vascular surgery	31 (4.4)
Prostate surgery	21 (3)
Circumcision	100 (14.1)
Testicular surgery	23 (3.2)
Vasectomy	79 (11.1)
Spinal surgery	17 (2.4)

HIV: human immunodeficiency virus; ^∗^continuous variables are expressed as median (range) and categorical variables as frequency (percentage).

**Table 2:** Stratified analysis of the ability to penetrate.

Examination		Adjunctive therapy	
		No	Yes
Basal	% patients with EHS ≥ 3	26.6% (21.3 to 31.8)	26.8% (22.7 to 31.0)
First examination (*n* = 710)	% patients with EHS ≥ 3	49.1% (43.1 to 55.0)	41.0% (36.4 to 45.6)
	Absolute difference^∗^	22.5% (14.6 to 30.4)	14.1% (7.9 to 20.3)
Second examination (*n* = 412)	% patients with EHS ≥ 3	36.7% (28.9 to 44.3)	38.5% (32.6 to 44.4)
	Absolute difference^∗^	10.1% (0.8 to 19.4)	11.7% (4.7 to 18.9)

^∗^Absolute difference in the percentage of patients able to penetrate, with a confidence interval of 95%, compared to baseline.
